# Efficacy and safety of rivaroxaban versus placebo after lower extremity bypass surgery: A post hoc analysis of a “CASPAR like” outcome from VOYAGER PAD

**DOI:** 10.1002/clc.23926

**Published:** 2022-10-17

**Authors:** Marc P. Bonaca, Michael Szarek, E. Sebastian Debus, Mark R. Nehler, Manesh R. Patel, Sonia S. Anand, Eva Muehlhofer, Scott D. Berkowitz, Lloyd P. Haskell, Rupert M. Bauersachs

**Affiliations:** ^1^ Department of Medicine, Division of Cardiology University of Colorado School of Medicine Aurora Colorado USA; ^2^ CPC Clinical Research Aurora Colorado USA; ^3^ Department of Vascular Medicine, Vascular Surgery – Angiology – Endovascular Therapy University of Hamburg‐Eppendorf Hamburg Germany; ^4^ Department of Surgery University of Colorado School of Medicine Aurora Colorado USA; ^5^ Duke Clinical Research Institute, Division of Cardiology Duke University Medical Center Durham North Carolina USA; ^6^ Population Health Research Institute, Hamilton Health Sciences McMaster University Hamilton Ontario Canada; ^7^ Bayer AG Research & Development Wuppertal Germany; ^8^ Janssen Research and Development Raritan New Jersey USA; ^9^ Department of Vascular Medicine, Klinikum Darmstadt, Darmstadt, and Center for Thrombosis and Hemostasis University of Mainz Mainz Germany

**Keywords:** antithrombotic therapy, CASPAR, lower extremity revascularization, peripheral artery disease, thrombosis, VOYAGER PAD

## Abstract

**Background:**

The Clopidogrel and Acetylsalicylic Acid in Bypass Surgery for Peripheral Arterial Disease (CASPAR) trial is the only large, double‐blind, placebo‐controlled trial of dual antiplatelet therapy (DAPT) versus aspirin in patients with peripheral artery disease (PAD) after lower extremity revascularization (LER). The trial was neutral for index‐graft occlusion/revascularization, amputation or death (hazard ratio [HR] 0.98, 95% confidence interval [CI] 0.78–1.23, *p* = .87) with an excess of global utilization of streptokinase and tissue plasminogen activator for occluded coronary arteries moderate or severe bleeding (HR 2.84, 95% CI 1.32–6.08, *p* = .007).

**Hypothesis and Methods:**

VOYAGER‐PAD demonstrated that rivaroxaban significantly reduces acute limb ischemia (ALI), major amputation, myocardial infarction (MI), stroke and CV death but increased bleeding. The relative efficacy and safety of rivaroxaban in a CASPAR like population and for similar outcomes is unknown. The current analysis is a post‐hoc exploratory analysis of a “CASPAR like” composite of ALI, unplanned index limb revascularization (UILR), amputation or CV death in surgical patients.

**Results:**

In the 2185 who underwent surgical LER, rivaroxaban reduced the CASPAR endpoint at 1 (HR 0.76, 95% CI 0.62−0.95, *p* = .0133) and 3 years (HR 0.84, 95% CI 0.71−1.00, *p* = .0461, Figure). There were similar reductions in composites of ALI, amputation or CV death (HR 0.79, *p* = .0228) and ALI, UILR, amputation, MI, IS or CV death (HR 0.85, *p* = .0410).

**Conclusions:**

The combination of rivaroxaban and aspirin significantly reduces ischemic outcomes in patients with PAD after LER. Although no formal head‐to‐head comparison exists, in a similar population and for similar outcomes, this regimen demonstrated benefit where trials of DAPT were neutral. These data suggest that factor Xa inhibition may provide specific benefits in this population and that DAPT should not be considered a proven substitution.

After lower extremity revascularization (LER), patients with peripheral artery disease (PAD) are at heightened thrombotic risk. Despite this risk, there are few randomized trials demonstrating the efficacy of antithrombotic therapies after LER. Clinicians caring for patients with PAD have largely adopted practices established in the context coronary revascularization where efficacy has been demonstrated primarily for the reduction of coronary complications.^1^

Recently, VOYAGER PAD (the vascular outcomes study of ASA along with rivaroxaban in endovascular or surgical limb revascularization for PAD) (www.clinicaltrials.gov NCT02504216) established the efficacy of low‐dose rivaroxaban with low‐dose aspirin for reducing thrombotic events of the limb, heart and brain in PAD after LER.^2^ The results have led to questions including whether they simply support “more intensive” antithrombotic therapy (e.g., dual antiplatelet therapy [DAPT]) or whether the results are specific to the combination studied. While the efficacy and safety of rivaroxaban, both in relative and absolute terms, was consistent whether added to aspirin alone or added to DAPT, there was no direct comparison of aspirin with rivaroxaban versus DAPT.^2^

In this context, the Clopidogrel and Acetylsalicylic Acid in Bypass Surgery for Peripheral Arterial Disease (CASPAR) trial is relevant.^3^ As the only large, double‐blind, placebo‐controlled trial of DAPT versus aspirin in patients with PAD after LER, it serves as reference point to address this question. CASPAR enrolled 851 patients undergoing lower extremity bypass and evaluated a primary composite of index‐graft occlusion/revascularization, above‐ankle amputation or death. Patients receiving low‐dose aspirin were randomly assigned to clopidogrel or placebo for 6−24 months. The trial was neutral at a median of 364 days (HR 0.98, 95% CI 0.78–1.23, *p* = .87) with a significant excess of global utilization of streptokinase and tissue plasminogen activator for occluded coronary arteries moderate or severe bleeding with DAPT (HR 2.84, 95% CI 1.32–6.08, *p* = .007).^3^

The surgical subgroup from VOYAGER demonstrated consistent efficacy for the composite of acute limb ischemia (ALI), major amputation of vascular etiology (amputation), myocardial infarction (MI), ischemic stroke (IS) or CV death (HR 0.81, 95% CI 0.67–0.88, *p* = .026).^4^ Although a direct comparison with CASPAR is not possible due to differences in standard of care over time, trial outcomes, and duration of follow‐up; evaluating the impact of rivaroxaban for a “CASPAR like outcome” offers insights as to the robustness of the VOYAGER PAD findings when considering previously proposed composite efficacy outcomes.^2^ We therefore performed a post hoc exploratory analysis of a “CASPAR like” composite of ALI, unplanned index limb revascularization (UILR), amputation or CV death in surgical patients at 1 year (to approximate median follow‐up in CASPAR) and total follow‐up. Additional analyses excluding UILR, including MI and IS, and restricting to bypass only were performed to evaluate for consistency. All patients provided informed consent and all relevant ethics approvals were obtained.

In the 2185 patients who underwent surgical revascularization, rivaroxaban reduced the CASPAR‐like endpoint at 1 year (HR 0.76, 95% CI 0.62−0.95, *p* = .0133) and 3 years (HR 0.84, 95% CI 0.71−1.00, *p* = .0461, Figure 1) with a placebo rate of 38.5/100 pt‐years and an absolute reduction of 6.5 events/100 pt‐years translating into a number needed to treat (NNT) of 16 at 3 years. There were similar significant reductions in composites of ALI, amputation or CV death (HR 0.79, *p* = .0228) and ALI, UILR, amputation, MI, IS or CV death (HR 0.85, *p* = .0410). In addition, when restricting to the 1448 treated with bypass, the composite of ALI, amputation, UILR or mortality was significantly reduced (*p* = .0481).

**Figure 1:**
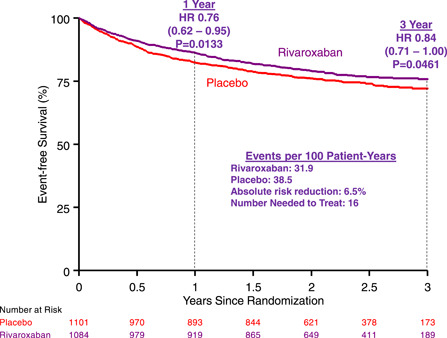
Freedom from the composite of acute limb ischemia, major amputation, unplanned index limb revascularization or cardiovascular mortality with rivaroxaban versus placebo over 3 years from randomization. Composite of acute limb ischemia, major amputation, unplanned index limb revascularization or CV mortality with rivaroxaban versus placebo. Absolute risk reduction and number needed to treat are shown for 3 years.

These results should be taken together with previously reported data in the surgical subgroup showing that rivaroxaban increased International society on thrombosis and haemostasis (ISTH) major bleeding in surgical patients (ISTH major HR 1.37, 95% CI 0.83–2.25, *p* = .89) with a number needed to harm of 83.^4^ In addition, rivaroxaban was associated with trends towards lower risk of CV death (HR 0.78, 95% CI 0.56 – 1.08) and all cause mortality (HR 0.86, 95% CI 0.67−1.12) versus antiplatelet therapy alone.^4^

The current analysis has important limitations including those inherent in cross trial comparisons and its post‐hoc exploratory nature. In this light the data should be viewed as a post hoc analysis within VOYAGER PAD to demonstrate consistency of benefit rather than a direct trial comparison. Results show a significant benefit for a CASPAR like outcome in surgical patients and a net benefit with a NNT of 16 and NNH of 83 for ISTH major bleeding consistent with the overall trial results. In addition, the observed benefit of rivaroxaban at 1 year where results with DAPT in CASPAR were neutral, suggests that mechanism and dose are more important than intensity as measured by number of agents alone when considering antithrombotic therapy for PAD. In addition, they suggest that thrombin inhibition may be particularly important in PAD where more intensive P2Y_12_ inhibition has not shown benefit.^5^

Clinicians caring for patients with PAD have, out of necessity, adopted antithrombotic strategies proven in other disease states and for other outcomes. The results of VOYAGER raise questions about how to consider DAPT relative to rivaroxaban. In similar patients and for similar outcomes, DAPT has not been shown to be beneficial, and a prospective randomized controlled trial comparing these strategies is unlikely. Therefore, in the near term for patients with PAD after LER, a strategy that incorporates rivaroxaban 2.5 mg has the strongest evidence for event reduction.

## CONFLICTS OF INTEREST

Dr. Bonaca reports receiving grant support from Amgen, AstraZeneca, Merck, Novo Nordisk, Pfizer, and Sanofi and was supported by the American Heart Association Strategically Focused Research Network in Vascular Disease under award numbers 18SFRN3390085 (BWH‐DH SFRN Center) and 18SFRN33960262 (BWH‐DH Clinical Project). The content is solely the responsibility of the authors and does not necessarily represent the official views of the American Heart Association. Dr. Szarek reports grant support from Resverlogix, Baxter, and Janssen; Personal fees from CiVi and Esperion; Grant support, personal fees, and nonfinancial support from Sanofi; and grant support and nonfinancial support from Regeneron; Dr. Debus, receiving grant support from Cook and Terumo Aortic; Dr. Nehler, being employed by CPC Clinical Research; Dr. Anand, receiving lecture fees from Bayer and Janssen; Dr. Patel, receiving grant support, advisory board fees, and consulting fees from AstraZeneca, grant support from Medtronic and Philips Healthcare, and grant support and advisory board fees from Heartflow; Dr. Muehlhofer reports being employed by Bayer; Dr. Berkowitz, being employed by Bayer; Dr. Haskell, being employed by Janssen Pharmaceuticals and owning stock in Johnson & Johnson; Dr. Bauersachs, receiving consulting fees and lecture fees from Bristol‐Myers Squibb, Daiichi Sankyo, and Pfizer.

## Data Availability

Data for this manuscript may be shared on reasonable request to the corresponding author.
